# Synthesis and Characterization of Porous Chitosan/Saccharomycetes Adsorption Microspheres

**DOI:** 10.3390/polym14112292

**Published:** 2022-06-05

**Authors:** Wei Song, Qingzhu Zhang, Yuxin Guan, Wanyan Li, Siyu Xie, Jin Tong, Mo Li, Lili Ren

**Affiliations:** 1 Key Laboratory of Bionic Engineering (Ministry of Education), College of Biological and Agricultural Engineering, Jilin University, Changchun 130022, China; song_wei20@mails.jlu.edu.cn (W.S.); guanyx1818@mails.jlu.edu.cn (Y.G.); liwy1818@mails.jlu.edu.cn (W.L.); xiesy1818@mails.jlu.edu.cn (S.X.); jtong@jlu.edu.cn (J.T.); 2 School of Engineering, Huzhou University, Huzhou 313000, China; zhangqz@zjhu.edu.cn

**Keywords:** chitosan, saccharomycetes, porous microspheres, adsorbents

## Abstract

Porous chitosan/saccharomycetes adsorption microspheres were successfully prepared by using silica gel as porogen. The morphology of porous chitosan/saccharomycetes microspheres was characterized by scanning electron microscopy, the interaction between molecules was characterized by Fourier transform infrared spectroscopy, and the crystallization property of the microspheres was characterized by X-ray diffraction. The results showed that the adsorption sites of amino and hydroxyl groups had been provided by the porous chitosan/saccharomycetes microspheres for the removal of preservatives, pigments, and other additives in food. The surface roughness of microspheres could be improved by increasing the mass ratio of saccharomycetes. The increase in silica gels could make the microsphere structure more compact. The porous chitosan/saccharomycetes microspheres could be used as adsorbents to adsorb doxycycline in wastewater.

## 1. Introduction

Antibiotics are widely used drugs, but the antibiotic pollution caused by their abuse has always been the focus of attention in the field of food safety testing [1]. As a common antibiotic drug, doxycycline has serious production pollution [2]. Antibiotics can enter the organism through food and interfere with normal physiological functions. Doxycycline is a semi-synthetic tetracycline antibiotic obtained by the deoxygenation of oxytetracycline, and its agent is hydrochloride. Its mechanism is the same as tetracycline, which mainly interferes with the protein synthesis of sensitive bacteria. Using doxycycline will stimulate the gastrointestinal tract, resulting in reflex vomiting, and other symptoms [3,4]. In addition to its medical application, doxycycline is also widely used in livestock, animal husbandry, and other breeding industries to prevent and treat animal diseases and as an additive to feed to promote the growth of livestock. However, the metabolic rate of organisms to antibiotics is very low, so a large number of unmetabolized antibiotics and metabolites that are not completely metabolized enter the natural environment with the excreta and the residual antibiotics in the environment cannot be decomposed and removed in time and effectively. The residual antibiotics in the natural environment are enriched in higher organisms and human bodies through the food chain. When the concentration exceeds the safe dose, it will seriously threaten the health of organisms and human beings. The maximum residue limit of veterinary drugs in animal food (GB 31650-2019, Announcement No. 235 of the Ministry of Agriculture, China) stipulates that the maximum residue limit of doxycycline (doxycycline) in poultry muscle is 100 μg/kg. Some natural raw materials in food contain biological toxins or produce biological toxins due to processing links, which directly threaten human life and health. For example, biogenic amine toxins mostly exist in fermented meat products, which easily induce colon cancer, rectal cancer, and so on.

Porous polymer materials have been gradually introduced into the field of food safety detection for the detection and analysis of a variety of substances and show many advantages, such as low detection limit, strong enrichment ability, good application effect, and so on. For example, a porous beta-cyclodextrin polymer was used as a new sorbent for the determination of bisphenol A, bisphenol F, and bisphenol AF in beverages [5]. Indium oxide nanoparticle-functionalized porous polymer has been applied to the determination of synthetic pigments in food samples, such as candy, jelly, jam, juice, and carbonated drinks [6]. However, as a new material, before it is put into practical application, porous polymer materials still have some problems, such as weak selectivity, inconvenient separation and detection after adsorption, and they are easily affected by the environment, and so on.

The mechanism of adsorption and detection of toxic or hazardous materials for adsorptions is the single mode of the reverse phase, chelation through functional groups, or ion exchange in the literature. However, the selectivity of the target material is not selective, which cannot eliminate the influence of impurity interference and matrix effect on the detection results. According to the structural characteristics of various toxic and harmful substances and from the perspective of the interaction mechanism between adsorbent and target substances, a mixed-mode adsorbent with a synergistic effect with target substances can effectively improve the enrichment selectivity of the target substances. As adsorption materials, compared with activated carbon and inorganic adsorbents, polysaccharide microspheres have unique advantages; they not only contain a large number of modifiable sites to provide anchor points for adsorption ligands but can also improve the adsorption performance further by hybridization with other heterogeneous components [7,8]. A uniform spherical morphology also allows the adsorbent to be easily assembled into the adsorption columns [9].

Chitosan, also known as deacetylated chitin, is obtained by the deacetylation of chitin [10], which is widely existent in nature. Chitin is a kind of linear polysaccharide, which maintains and protects biological bodies and crustaceans, and is abundant in cuticles of invertebrates and fungal cell walls [11]. Under specific conditions, chitosan can undergo chemical reactions such as hydrolysis, alkylation, acylation, carboxymethylation, sulfonation, nitration, halogenation, oxidation, reduction, condensation, and complexation. The chitosan molecular chain contains many functional groups, such as amino groups and hydroxyl groups. These groups are more active, especially the N atom in the amino group that contains lone-pair electrons, which easily react with hydrogen ions. Acylation modification can be realized by mixing with derivatives of some organic acids (anhydride, acyl halide, etc.) and introducing aliphatic or aromatic acyl groups. An acylation reaction can occur on the hydroxyl group (O-acylation) to generate an ester or amino group (n-acylation) to generate amide. Chloroalkanoic acid or glyoxylic acid can react with hydroxyl or amino groups on chitosan to obtain the corresponding carboxylated chitosan derivatives [12]. The amino group and the hydroxyl group in the chitosan have an alternative to electron and empty orbital, and the amino protonated cation can be electrostatically bonded to a strong mymeousromycin present in an anionic state. Chitosan and its derivatives, which have good adsorptivity and are very economical, are popular adsorptive materials that people have explored in recent years [13].

The microbial surface is rich in chemical functional groups, such as carboxyl, amino, hydroxyl, etc. Saccharomycetes cells are no exception; saccharomycetes, as a natural adsorbent raw material, were widely used in wastewater adsorption treatments [14]. The newly prepared porous carbon saccharomycetes have shown broad application potential. The main reason was that the porous saccharomycetes carbon had a developed pore structure, highly-specific surface area, and a good adsorption capacity [14,15]. However, in practical applications, the small size of the saccharomycetes particles caused a series of problems, such as the agglomeration of the adsorbent, the blockage of equipment, difficulty in solid–liquid separation, and so on.

Chitosan and saccharomycetes (yeast) are two kinds of materials with high adsorption capacity, so their different combination and preparation methods are worthy of study. There are many functional groups, such as the hydroxyl group, amino group, carboxyl group, phosphoryl group, and so on, on the cell wall surface of saccharomyces, which make saccharomyces play an important role in the application of adsorption materials. Chitosan/saccharomycetes hybrid hydrogel beads were fabricated by the facile introduction of saccharomycetes cells into the chitosan matrix through alkali gelation, and these are good candidates for controlled-release carrier biomaterials [16]. Double-layer alginate beads coated with chitosan were constructed for the entrapment of saccharomycetes cells used in the fermentation of alcohol [17]. Saccharomycetes spores could serve as an efficient carrier of chitosan microspheres for intracellular delivery [18]. By introducing the protective layer and maintaining the cell viability, the active saccharomycetes cells were coated with multilayer films composed of antimicrobial chitosan. In addition, the multilayer coating was degraded by a chitosan enzyme, which would help to expand the application of various antimicrobial materials in cell surface modification [19]. Saccharomyces cerevisiae cells have been immobilized onto microwave-synthesized magnetic chitosan microparticles, which can be successfully used for sucrose hydrolysis, hydrogen peroxide decomposition, and the adsorption of important xenobiotics, e.g., dyes [20].

The pore structure hinders the transport of adsorbed ions in a liquid-phase solution in a porous adsorbent, and the pore structure can reduce its diffusion resistance and increase the contact sites between the materials and the adsorbates. At the same time, the porous structure increased the diffusion channel of the adsorbed substance into the microsphere, enhanced its swelling and adsorption rate, and enabled the material to quickly adsorb various pollutants in the aqueous solution, thus greatly improving the purification effect of the adsorbent on the adsorbed wastewater [21]. However, there were no reports about the preparation of porous chitosan/saccharomycetes microspheres aimed at green applications and environmental protection.

There are many methods to prepare chitosan-based microspheres, including cross-linking with anions [22], precipitation [23], complex-coacervation [24], modified emulsification and ionotropic gelation [25], precipitation-chemical cross-linking [26], glutaraldehyde cross-linking [27], thermal cross-linking [28], ball-dropping addition, and more. In this paper, the ball-dropping addition method was chosen. Because chitosan is acid-soluble and alkali-insoluble, this property is applied by the ball-dropping addition method. Titration was considered to be the simplest method of spheronization, and the prepared microspheres are usually millimeters in size. Under the action of pressure or gravity, the polysaccharide solution was ejected from the nozzle, and spherical droplets were formed under the action of surface tension, which finally fell into the coagulation bath for solidification and molding. In order to increase the specific surface area of the microsphere, more adsorption sites were obtained, and therefore, silica gel was used herein. Silica gel has been used as a porogen in many preparations of porous materials, such as microporous chitosan membranes [12,29,30,31] and chitosan microspheres [32]. According to the property that silica gel can be dissolved in hot alkaline solution, silica gel was evenly mixed in chitosan saccharomycetes solution, and then the ball was added dropwise. The porous formation is due to the dissolution of silica gel in an alkaline medium [30]. The materials used in this method were simple, easy to operate, and had a uniform void distribution. Therefore, in order to form porous chitosan-based microspheres with stable physicochemical properties and excellent adsorption ability, the study of the preparation and the characterization of porous chitosan/saccharomycetes are very important.

The purpose of this paper was to prepare porous composite microspheres with chitosan, saccharomycetes, and silica gel and then characterize the appearance and structure of the porous chitosan/saccharomycetes microspheres by scanning electron microscopy, Fourier-transform infrared spectroscopy, and X-ray diffraction and measure the porosity of the porous microspheres. By changing the mass ratio of chitosan, saccharomycetes, and silica gel, the effects of different mass ratios on the sphericity and porosity of the microspheres were studied, and the best mass ratio of the microspheres was determined. The combination of chitosan and saccharomycetes avoids many of the problems caused by saccharomycetes particles that are too small and also gives full play to the adsorption capacity of the porous chitosan/saccharomycetes microspheres.

## 2. Materials and Methods

### 2.1. Materials

Chitosan (the degree of deacetylation is 88.0%, the molecular weight is 161.16 kDa, and the viscosity is 51 MPa·s) and silica gel (200–300 mesh) were supplied by the Sinopharm Chemical Reagent Co. Ltd. No. 20120330 (Shanghai, China). The saccharomycetes were obtained from the Angel saccharomycetes Co., Ltd (Yichang, Hubei Province, China). Sodium hydroxide (NaOH). Ethanol and acetic acid (36%) were obtained from the Beijing Beihua Fine Chemicals Co. Ltd. (Beijing, China). All of these materials were used as received without further purification.

### 2.2. Preparation of Porous Chitosan/Saccharomycetes Microspheres

The preparation of porous chitosan/saccharomycetes microspheres is mainly divided into two steps: the synthesis of hydrogel beads mixed with chitosan, saccharomycetes, and silica gels, and the formation of porous chitosan/saccharomycetes microspheres (including the dissolution of silica gel in hydrogel beads and the pre-freezing and freeze-drying process of porous beads).

First, 3 g of chitosan powder was dissolved in 100 mL of 0.2 M acetic acid solution to form a homogeneous chitosan solution with 3% (*w*/*v*) as a dispersed phase. The resulting chitosan solution was mixed with 6 g of saccharomycetes and then put into a water bath at 60 °C for 6 h. Then different amounts of silica gels (1, 2, 3, and 4 times the mass of the chitosan) were added into the mixture solution of chitosan and saccharomycetes with a pH of 5.1. Then, the mixed solution was continuously stirred at 25 °C for 4 h and then vibrated by using a KQ-600KDE ultrasonic cleaner (Kunshan, China) with 480 W of power for 15 min to remove the air bubbles.

The obtained solution was injected as monodispersed droplets into 100 mL of 1.0 M NaOH aqueous solution through the use of a syringe pump (LSP02-1B, Longer Precision Pump Co., Ltd., Changchun, China) with a microneedle with a diameter of 500 μm to form the hydrogel beads. Then the hydrogel beads were placed into 100 mL of 1.0 M NaOH aqueous solution at 80 °C for 2 h to dissolve the silica gel to form the porous beads. The yielded porous beads were repeatedly washed with deionized water until neutral and then frozen in a deep cooler at −20 °C for 8 h. The frozen beads were dehydrated in a YTLG-12A freeze dryer (Shanghai Yeto Technology Co., Ltd., Shanghai, China) at −60 °C to obtain chitosan/saccharomycetes microspheres with a porous structure.

### 2.3. Characterization of Porous Chitosan/Saccharomycetes Microspheres

#### 2.3.1. Fourier Transform Infrared Spectroscopy (FT-IR)

The chitosan/saccharomycetes microspheres were characterized with FT-IR (Nicolet IS50, Madison, Wisconsin, USA) in the wavelength range of 500–4000 cm^−1^. The samples were prepared by grinding the samples together with KBr and then pressed into a disc. The resolution was 0.1 cm^−1^, and the total number of scans was 32.

#### 2.3.2. Scanning Electron Microscopy (SEM)

The microspheres’ size and the surface morphologies of the chitosan/saccharomycetes microspheres were tested by a ZEISS EVO 18 field-emission scanning electron microscope (Zeiss, MERLIN Compact, Jena, Germany) after coating with a thin layer of gold with the help of an FPMRC-SPT-20 gold sputter (Felles Photonic Instruments, Shanghai, China) with a relative sputtering rate of 1.0.

#### 2.3.3. Porosity

The porosities of the porous chitosan/saccharomycetes microspheres were measured using a liquid displacement method according to the method reported by Ren et al. [33]. Five measurements were taken for each sample.

#### 2.3.4. X-ray Diffraction (XRD)

The structures of the porous chitosan/saccharomycetes microspheres were characterized by using a Smartlab 9 kW X-ray diffractometer (Rigaku, Tokyo, Japan) with 9 kW radiation at 45 KV and 200 mA. The XRD was recorded over the angle of 5–70° at a speed of 4°/min.

#### 2.3.5. Zeta Potential Analysis

The zeta potential measurements of the samples were measured by dynamic light scattering (DLS) using a Malvern Zetasizer Nano-ZS90 (Malvern Instruments Ltd., Malvern, UK). The intensity of the scattered light was detected at 90° to the incident beam. The measurements were performed using the samples prepared by diluting 0.2 M chitosan-based acetic acid solution at 25 °C in the pH range of 2.0–10.0.

#### 2.3.6. Adsorption Experiment

In order to determine the adsorption capacity of the porous chitosan/saccharomycetes microspheres for doxycycline, an adsorption experiment was performed as follows. An aqueous solution of doxycycline with an initial concentration (*C*_0_) of 20 mg/L and a pH of 8.0 was obtained. Then, 0.1 g/L porous chitosan/saccharomycetes microspheres were added to the doxycycline solution, which was placed in a water-bath shaker under 25 °C and at100 rpm. A fixed volume of doxycycline solution was taken out at different contact times (0–150 min) and analyzed at 352 nm using a UV spectrophotometer (Ruili Analytical Instrument Company, Beijing, China) to find the concentration of doxycycline (*C_t_*, mg/L). Five measurements were taken for each sample. The adsorption capacity was calculated according to the following equations:

(1) $$q_{t}=\frac{\left(c_{0}-c_{t}\right)\times V}{m}$$

where *q_t_* is the adsorption capacity (mg/g), *m* is the weight of the microspheres added to the doxycycline solution (mg), *V* is the volume of the doxycycline solution taken out for every doxycycline concentration analysis (L).

#### 2.3.7. Statistical Analysis

The differences between the factors and levels in this work were evaluated by the analysis of variance (ANOVA). Duncan’s multiple range tests were used to compare the means of all of the data to identify which groups were significantly different from other groups (*p* < 0.05). Moreover, the data in this work are presented as mean ± standard deviation.

## 3. Results and Discussion

### 3.1. Formation of Porous Chitosan/Saccharomycetes Microspheres

**Figure 1:**
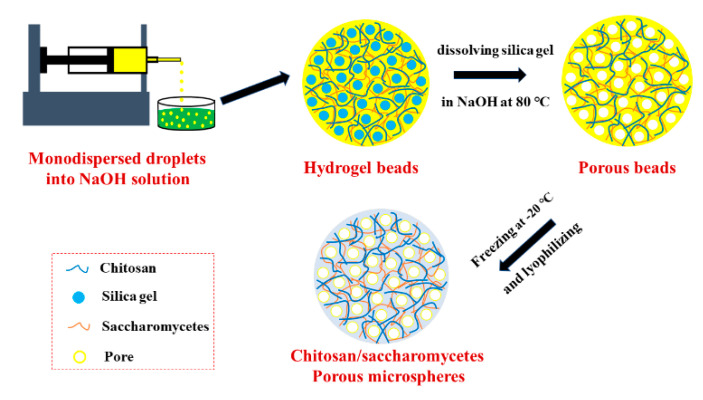
The schematic illustration of porous chitosan/saccharomycetes microspheres preparation process.

The formation process of porous chitosan/saccharomycetes microspheres was as shown in Figure 1. The natural chitosan molecule has a linear structure. Because of hydrogen bonding and van der Waals force, chitosan molecules usually show a network structure formed by interweaving. Chitosan is insoluble in a basic medium, but if the pH of the solution is adjusted to be acidic, the amino groups on chitosan molecules can be protonated, and electrostatic repulsion will be generated between the molecules. As a result, the chitosan molecules will become longer and better dispersed in the aqueous solution [16], and the chitosan acid solution will enter into a transparent gel state. When the saccharomycetes was introduced into the chitosan acid solution, saccharomycetes formed a saccharomycetes–chitosan molecular complex with chitosan molecules in the solution through the hydrogen bonding between -NH_2_ and -OH groups, which is shown by the FTIR results in Section 3.2. In addition, the cell wall of saccharomycetes is similar to the plant epidermis and has a considerable natural tensile strength to prevent serious shrinkage, which would alleviate the serious shrinkage of chitosan hydrogels. More importantly, the unique, spherical hollow space inside the saccharomycetes cells might serve as a small reservoir to accumulate more water, which is conducive to enhancing the water absorption and the preservation of the chitosan-based composite hydrogels.

Then, silica gel was added to form a uniform dispersion. Then, the dispersed phase was separated into monodisperse droplets by the shearing forces of the continuous phase in the syringe needle. At the same time, when it was dropped into NaOH, the droplets were solidified by acid–base neutralization; that is, the amino groups of the chitosan molecules on the saccharomycetes–chitosan complex were deprotonated so that the chitosan molecules lost their protocolloid state [16,34] and recovered their network structure, thus forming saccharomycetes–chitosan precursors. The saccharomycetes–chitosan precursors are macroscopically spherical, with uniform size distribution, and are milky yellow in color. The milky yellow color is due to the addition of saccharomycetes. In addition, silica gel was chosen as the pore-making agent due to the characteristic that silica gel is insoluble in water but soluble in hot sodium hydroxide solution. Before dropping the ball, the silica gel was added into the chitosan–saccharomycetes solution, which was evenly distributed by stirring. Because titration is to drop the solution into sodium hydroxide solution, sodium hydroxide solution can be used to complete the preparation of the pores at the same time. In this way, the requirements for the hole-making environment and equipment were greatly reduced, the operation steps were simplified, the raw materials were simple and cheap, and the preparation cost was saved. Silica gel (xSiO_2_·yH_2_O) exists in the precursor, and when it exists in sodium hydroxide solution at 80 °C, silica gel reacts with NaOH; that is, at high temperatures, NaOH will separate out OH^−^, and the silica gel reacts with OH^−^ to generate H_2_O. Silica gel in the microspheres was removed by the reaction, thus forming porous microspheres. Then the porous chitosan/saccharomycetes microspheres were prepared by the simple procedure of freezing the porous microspheres and subsequent lyophilizing.

### 3.2. FTIR Analysis

**Figure 2:**
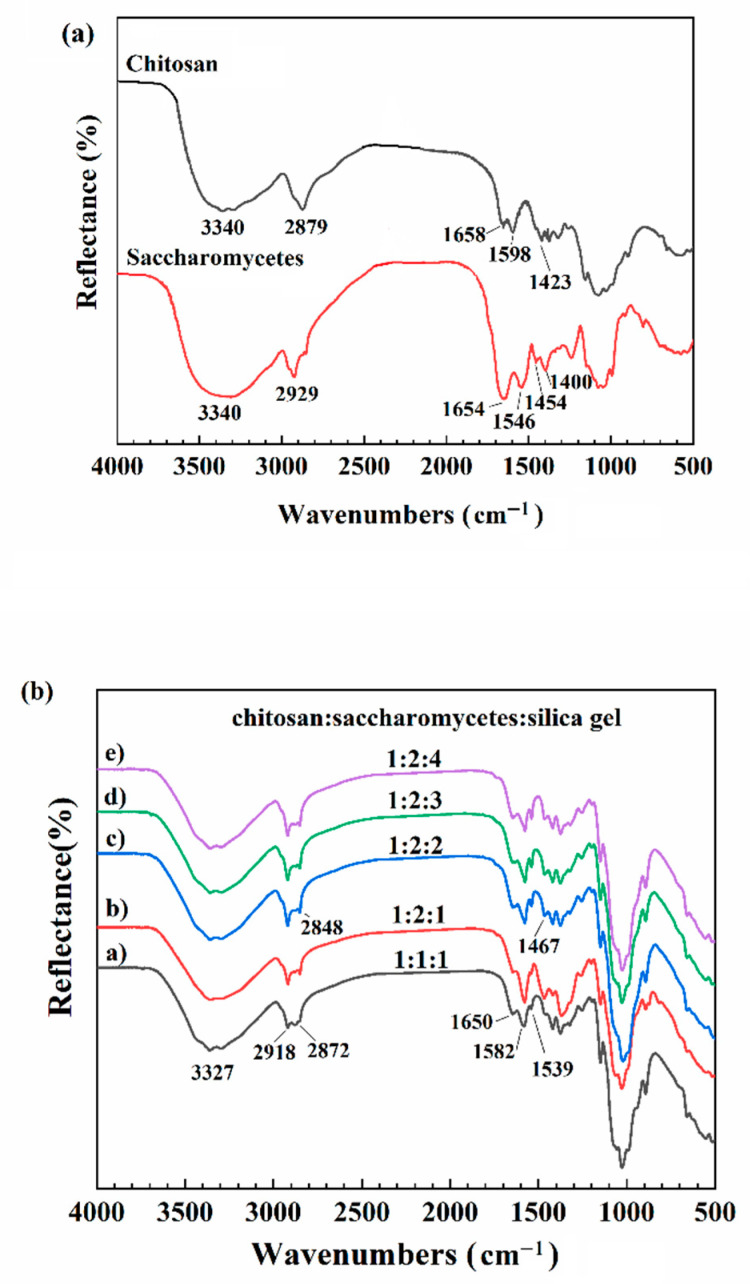
FTIR spectra of chitosan powder and saccharomycetes powder (**a**), and porous chitosan/saccharomycetes microspheres with different ratio of chitosan to saccharomycetes and silica gel (**b**).

In order to study whether the chitosan and saccharomycetes react and whether the silica gel in the microsphere is completely removed, the microspheres were subjected to FTIR. The infrared spectrum of composite microspheres is shown in different mass ratios in Figure 2.

As can be seen from Figure 2a, the FTIR spectrum of the chitosan powder had a strong peak around 3340 cm^−1^ due to the stretching vibration of O–H superimposed on the N–H stretching [35]. The peak at 2879 cm^−1^ was attributed to a -CH stretching vibration. The peaks at 1658, 1598, and 1423 cm^−1^ correspond to C=O stretching, N–H bending, and -OH bending vibrations, respectively [35,36,37]. For saccharomycetes, a wide and strong peak was observed at 3340 cm^−1^ due to hydroxyl-stretching vibrations. The peaks at 1654, 1546, and 1400 cm^−1^ were due to C=O in amide I, N-H in amide II, and C-N in amide III, respectively. Moreover, the bands in the region of 1200–1000 cm^−1^ were attributed to the stretching vibration of the C–O–C ring vibrations of carbohydrates [16,34].

The infrared spectrum of the porous chitosan/saccharomycetes microspheres was analyzed by the infrared spectrum of chitosan and saccharomycetes. In the FTIR spectrum of porous chitosan/saccharomycetes microspheres in Figure 2b, the above characteristic peaks of chitosan and saccharomycetes were almost all retained or stronger. However, most characteristic peaks have shifted. The peaks produced by O–H and N–H stretching vibration were much wider and stronger than those in single chitosan or saccharomycetes, which indicated that the chitosan network and saccharomycetes in porous chitosan/saccharomycetes microspheres were intertwined mainly through the strong intermolecular hydrogen bonding interactions between -NH_2_ and -OH groups, which helped to form a stable three-dimensional network [16]. The FTIR spectrum of the porous chitosan/saccharomycetes microspheres was very similar to previous findings [16]. It can be seen that by changing the ratio of chitosan, saccharomycetes, and silica gel, the FTIR spectra (Figure 2b) of the composite microspheres were not significantly affected. There was a strong peak near 3327 cm^−1^ because the oxygen stretching vibration was formed, and the -OH telescopic vibration peak, 3293 cm^−1^, was a -NH telescopic absorption peak formed by the tensile vibration of nitrogen, indicating that the multi-aperture glycan saccharomycetes composite formed [38,39,40]. The C–H telescopic characteristics of -CH_3_ and -CH in the microspheres are located at 2918 cm^−1^ and 2872 cm^−1^ [41,42], respectively. In addition, typical peaks at 1650, 1582, and 1539 cm^−1^ are caused by amide I, amide II, and amide III [23,25,27,28]. As the ratio of chitosan and silica gel ranged from 1:1 to 1:2, the peak located at 2872 cm^−1^ moved to 2848 cm^−1^, which was due to two keys in the carbon triple, and the -CH was changed into -CH_2_. The peak intensity at 1467 cm^−1^ became larger, which could be verified that the peak intensity estimated the relative content of -CH_2_.

### 3.3. Morphology and Porosities of Porous Chitosan/Saccharomycetes Microspheres

**Figure 3:**
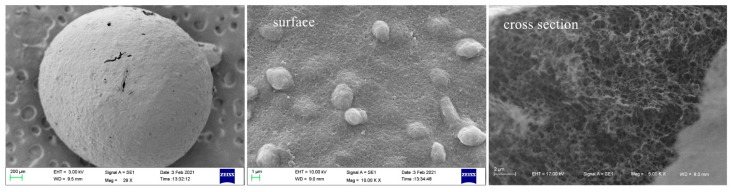
SEM images of the whole, surface, and cross-section of porous chitosan/saccharomycetes microspheres prepared with the mass ratio of chitosan to saccharomycetes and silica gel of 1:2:3.

**Figure 4:**
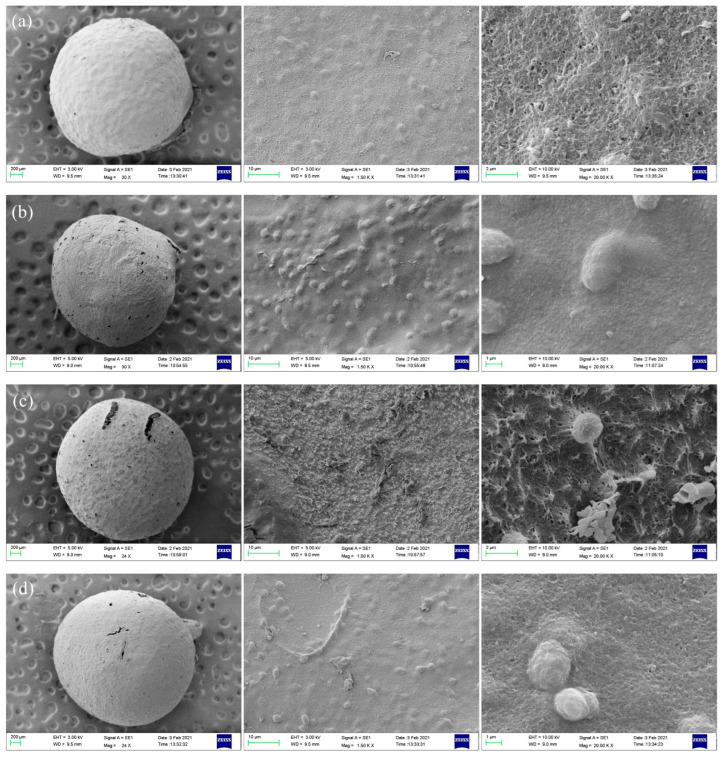

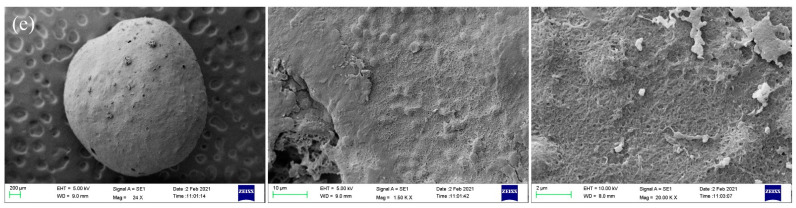
SEM images of the porous chitosan/saccharomycetes microspheres with different ratio of chitosan to saccharomycetes and silica gel of 1:1:1 (**a**), 1:2:1 (**b**), 1:2:2 (**c**), 1:2:3 (**d**) and 1:2:4 (**e**).

Figure 3 shows the SEM images of the whole, surface, and cross-section of the porous chitosan/saccharomycetes microspheres prepared with the mass ratio of chitosan to saccharomycetes and silica gel of 1:2:3; it can be observed that the porous formation, due to the dissolution of silica gel in alkaline medium, is evidenced at the surface and interior of the microspheres, and the pore diameters were uniform and about 200 ± 10 nm. Figure 4 shows the morphological structure of the porous chitosan/saccharomycetes microspheres prepared by different mass ratios of chitosan, saccharomycetes, and silica gel. From the overall figure, it can be seen that the diameter of porous chitosan/saccharomycetes microspheres ranged from 1.8 to 2.5 mm. Chitosan, as a molding adhesive, successfully played a coupling role between saccharomycetes.

Figure 4a shows the porous chitosan/saccharomycetes microspheres prepared with the mass ratio of chitosan to saccharomycetes and silica gel of 1:1:1. In Figure 4a, the microspheres are spherical with few pores, and the morphology of the microsphere surface is at 1500-times magnification. It can be seen that the microsphere surface was smooth with fewer protrusions. The morphology of the microsphere surface at 3000-times magnification, from which it can be seen that the microsphere surface is spongy and loose. While, in the literature, chitosan/saccharomycetes hybrid hydrogel beads fabricated by the facile introduction of saccharomycetes cells into the chitosan matrix through alkali gelation displayed a rugged plane with many saccharomycetes cells embedded in the chitosan network, and the chitosan/saccharomycetes microspheres still retained their original shape in contrast with their parent saccharomycetes cells. Some saccharomycetes cells aggregated closely and were deeply embedded in the crosslinked chitosan network [11].

Figure 4b shows the porous chitosan/saccharomycetes microspheres prepared by chitosan, saccharomycetes, and silica gel with a mass ratio of 1:2:1. The spheroidization of the microspheres was poor, and the pores were obvious. It can be seen from Figure 4b that the surface of the microspheres was rough and had many protrusions. Furthermore, the protrusions were evenly distributed, and the surface protrusion of the microspheres was more prominent. Figure 4c shows the porous microspheres prepared by chitosan, saccharomycetes, and silica gel with a mass ratio of 1:2:2. The microspheres have good sphericity, and it can be clearly seen that there are holes on the surface.

The surface of the microspheres was rough, while it can be seen from Figure 4c that there were protrusions on the surface of the microspheres. Figure 4d showed the porous microspheres prepared by chitosan, saccharomycetes, and silica gel with a mass ratio of 1:2:3. The microspheres had good spheroidization, and there were visible holes on the surface. It can be seen that the surface of the microspheres was rough, there were many protrusions, and the surface structure of the microspheres was compact and reticular fibrous structure. Figure 4e shows the porous microspheres prepared by chitosan, saccharomycetes, and silica gel with a mass ratio of 1:2:4. The microsphere was a little shriveled. It can be seen from Figure 4e that there were overlapping films on the surface of the microspheres. It can be seen that the surface of the microspheres was too tight, resulting in the presence of a detached film on the surface of the microspheres.

The protrusions on the surface of the microspheres were particles of saccharomycetes, by comparing two kinds of microspheres in Figure 4a,b. There were fewer protrusions on the surface of the microspheres; there were many protrusions evenly distributed in microspheres. Namely, the distribution of saccharomycetes was higher in Figure 4b. Furthermore, saccharomycetes had adsorption capacity, which can increase the adsorption capacity of microspheres [43].

In conclusion, in the case of the same mass ratio of chitosan and silica gel, by increasing the mass ratio of saccharomycetes, the content and distribution of saccharomycetes in microspheres can be improved so as to improve the adsorption capacity of microspheres, which could be proven in Section 3.5. Moreover, in Figure 4a, the surface of the microspheres was smooth with fewer pores, and the surface of the microspheres was rough with more pores. Because the surface roughness and pore structure can effectively increase the contact site between the microspheres and the adsorbate and increase the diffusion channel of the adsorbate into the microspheres, thus greatly improving the adsorption and purification effect of the microspheres on wastewater; that is to say, the mass ratio of the microsphere in Figure 4b was more suitable for making porous microsphere.

For the microspheres in Figure 4, that is, when the mass ratio of chitosan and saccharomycetes was the same, by changing the mass ratio of silica gel, the pore sizes of microspheres were increased with the increase in silica gel content. The average pore diameters of the porous chitosan/saccharomycetes microspheres with different mass ratios of chitosan to saccharomycetes and silica gel of 1:1:1, 1:2:1, 1:2:2, 1:2:3, and 1:2:4, were 184 ± 8, 175 ± 9, 192 ± 13, 200 ± 10 and 210 ± 15 nm, respectively. The surface holes of the balls with these three mass ratios were obvious, and the surface roughness of the microspheres was also good. However, in Figure 4e, the microsphere was a little shriveled. The spheroidization of the microspheres was poor, and the structure was too tight to prepare microspheres.

**Figure 5:**
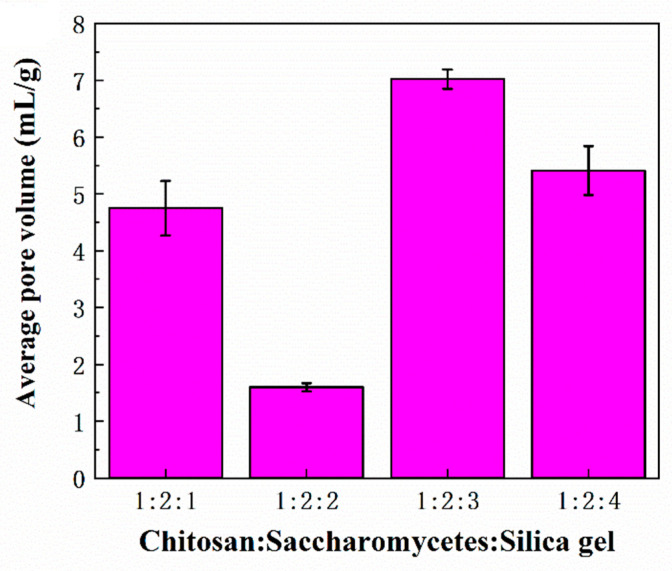
Average pore volume of the porous chitosan/saccharomycetes microspheres with different ratios of chitosan to saccharomycetes and silica gel.

**Figure 6:**
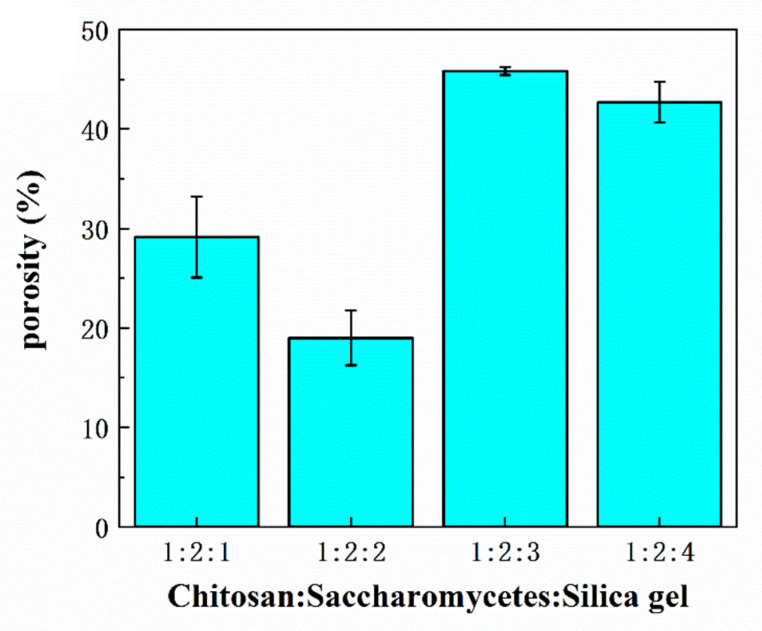
Porosity of the chitosan-based porous chitosan/saccharomycetes microspheres with different ratio of chitosan to saccharomycetes and silica gel.

Figure 5 and Figure 6 show the average pore volume and porosity of the porous chitosan/saccharomycetes microspheres with different mass ratios of chitosan to saccharomycetes and silica gel of 1:1:1, 1:2:1, 1:2:2, 1:2:3, and 1:2:4, respectively. According to Figure 5, among these kinds of microspheres, the porosity of the microspheres with chitosan, saccharomycetes, and silica gel of 1:2:3 was the highest. Furthermore, the higher the porosity, the stronger the adsorption capacity of the microspheres [44]. At the same time, it can be concluded from Figure 6 that the average pore volume (d) of the microspheres with chitosan, saccharomycetes, and silica gel of 1:2:3 among these microspheres, is also the largest. That is, the pore-forming property of the microspheres with chitosan, saccharomycetes, and silica gel of 1:2:3 is also the best. It could be found that when the mass ratio of silica gel increased, the porosity of the microspheres did not increase. The reason was that the silica gel in the surface layer of the chitosan/saccharomycetes hydrogel beads was easier to dissolve in hot sodium hydroxide solution, and the inappropriate mass ratio of chitosan and saccharomycetes: silica gel in the hydrogel beads would affect the generation of pores. At the same time, measuring the porosity of microspheres by the liquid exchange method can not ensure that all the pores inside the microspheres are filled with liquid, resulting in porosity error. However, the porosity measured by this method does not affect the analysis of the microsphere adsorption experiment because the microsphere adsorption experiment is also in the liquid environment, and the dense structure on the surface of the microsphere and the irregular distribution of the internal pores will prevent the liquid from entering the deeper interior of the microsphere. Therefore, the most suitable mass ratio for the preparation of porous chitosan/saccharomycetes microspheres was the mass ratio of chitosan: saccharomy: silica gel of 1:2:3.

By comparing the porosity of the porous chitosan microspheres prepared by other methods in the literature [45], one of the works focused on the porosity of a coal mine drainage sludge (CMDS)-beaded adsorbent made of chitosan and alginate, which was optimized by adding NaHCO_3_ powder to generate CO_2_ gas during the preparation process. The beaded adsorbents prepared by this method had good porosity and specific surface area, which were 16.8 and 21.2 m^2^g^−1^, respectively. However, the porosity of the porous chitosan/saccharomycetes microspheres prepared by the mass ratio of 1:2:3 was the highest, and the porosity and sphericity were also excellent. Porosity plays an important role in porous microspheres. For porous microspheres, the higher the porosity, the better the adsorption performance. Therefore, porous microspheres with better porosity were prepared according to the mass ratio and the preparation methods of these materials.

### 3.4. XRD Analysis

The crystallinity of the adsorbents plays an important role in the swelling performance, mechanical properties, and acid-base resistance, which affects the service life of adsorbents [35]. XRD has been widely used to reveal the characteristics of the crystalline structure of chitosan-based microspheres and to verify if any alteration of their crystallinity occurred upon the preparation of porous materials with different components.

**Figure 7:**
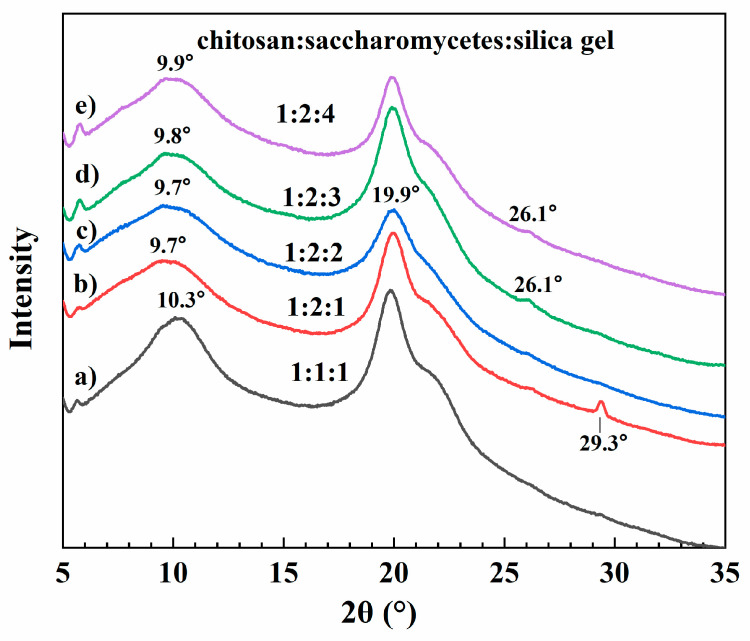
XRD pattern of the porous chitosan/saccharomycetes microspheres with different ratio of chitosan to saccharomycetes and silica gel of 1:1:1 (**a**), 1:2:1 (**b**), 1:2:2 (**c**), 1:2:3 (**d**), and 1:2:4 (**e**).

In order to determine the components of the porous chitosan/saccharomycetes microspheres and the effect of the content ratio on the crystal structure of the microspheres, the XRD analyses of the microspheres are depicted in Figure 7. The porous chitosan/saccharomycetes microspheres presented a narrow peak, which appeared at 2θ = 19.9°, and a broad peak around 10.3°, which were different from the characteristic peaks of chitosan and saccharomycetes [26]. The formation process of porous chitosan/saccharomycetes microspheres would change the crystallinity of both chitosan powder and saccharomycetes powder, which can be explained by the dissolution of chitosan in an acidic environment, and the expansion of the chitosan molecular chain due to electrostatic repulsion and the weak hydrophobic effect [35,37]. The peak around 19.9° and the shift of peak at 10.3 to lower 2θ with the increase in saccharomycetes contents proved that the intermolecular hydrogen bonding between chitosan and saccharomycetes limited the movement of the molecular chain segments and inhibited the crystallization process [46,47].

### 3.5. Application of Porous Chitosan/Saccharomycetes Microspheres

Zeta potential is one of the most important parameters, which is an important index to characterize the stability of the colloidal dispersion system and can determine the surface charge of the adsorbent. Zeta potential is a measure of the strength of mutual repulsion or attraction between particles. In practical operation, the change of the zeta potential of the adsorbent can provide a basis for the application of an adsorption method in the treatment of food additives.

**Figure 8:**
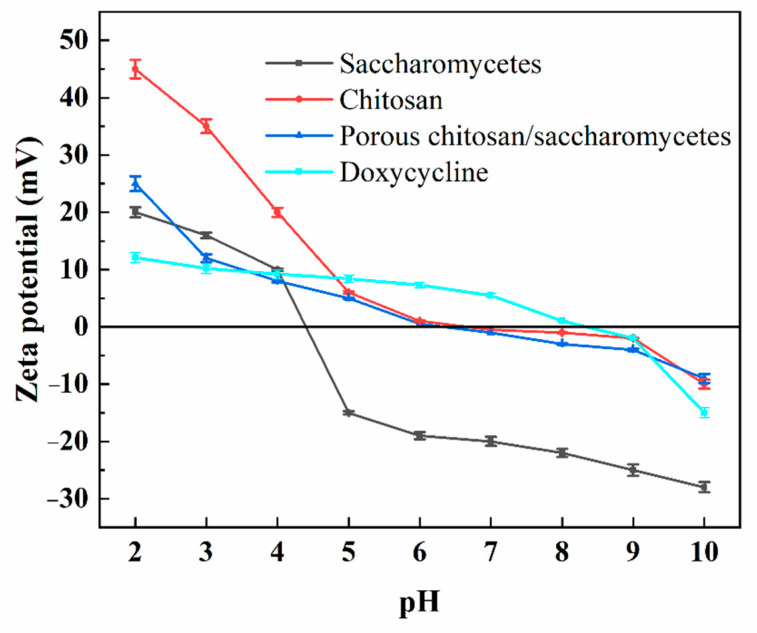
Zeta potential of saccharomycetes, chitosan, doxycycline and the porous chitosan/saccharomycetes microspheres with the ratio of chitosan to saccharomycetes and silica gel of 1:2:3.

Figure 8 shows the zeta potential of saccharomycetes, chitosan, porous chitosan/saccharomycetes microspheres, and doxycycline. The surface of the porous chitosan/saccharomycetes microspheres was positively charged under acidic conditions and negatively charged under alkaline conditions. In the zeta-potential analysis, the zero-charge point is often taken as the equilibrium state. When the zeta potential of porous chitosan/saccharomycetes microspheres was zero, the pH value was 6.7. The changes in the zeta potential of the porous microspheres and doxycycline were measured to provide a theoretical basis for the follow-up study of the adsorption mechanism of doxycycline removal by porous microspheres.

The porous chitosan/saccharomycetes microspheres prepared in this work could be used as adsorbents to adsorb and detect antibiotics and heavy metal ions in wastewater, and the additives and volatiles in food, such as the antibiotics used in livestock and milk and colorants in candy and beverages to ensure the quality of food and prolong the shelf life of food. For example, those chitosan derivatives that are used as a fruit juice clarifier; their clarification mechanism is related to the positive charge of chitosan molecules and can adsorb the negatively charged pectin, protein, and other substances in fruit juice. Due to the long-chain linear structure of chitosan, it can adsorb many particulate impurities on the same molecule and form flocs, which will then settle to achieve the purpose of clarification. In these applications, the possible contamination of food raw materials with saccharomycetes cells should be taken into account. First, the FTIR results showed that the chitosan network and saccharomycetes in the porous chitosan/saccharomycetes microspheres were intertwined, which proved that the existence of saccharomycetes isolated in food was almost impossible. In addition, even if there are, there are fewer saccharomycetes in food than there are bacteria, and they grow slowly, so they are less likely to become the dominant microbes in the food’s microbial community. Most saccharomycetes have a low decomposing activity in regards to protein and fat, so they are not the direct cause of food deterioration. Except the gas produced by alcohol fermentation causes the container to expand and crack, or red saccharomycetes are reproduced on the food surface, the saccharomycetes pollution of food is generally not obvious, and there is no actual damage.

In the preliminary work, in order to determine the adsorption capacity of the porous chitosan/saccharomycetes microspheres for an antibiotic, the adsorption test of doxycycline was carried out. It can be seen from Figure 8 that when the pH ranged from 6 to 8, electrostatic attraction occurred due to the heterogeneous charge of porous microspheres and doxycycline, which is conducive to the adsorption reaction.

**Figure 9:**
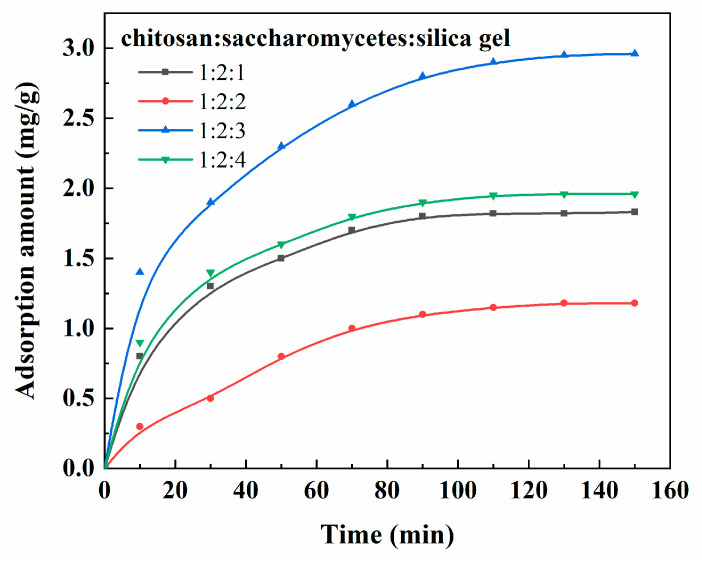
Adsorption property of the porous chitosan/saccharomycetes microspheres with the ratio of chitosan to saccharomycetes and silica gel for doxycycline.

The results in Figure 9 show that the porous chitosan/saccharomycetes microspheres showed a strong adsorption capacity for doxycycline. This was because there were lone-pair electrons and empty orbitals on the amino and hydroxyl groups of chitosan, and the cations formed by amino protonation could electrostatically bind to anionic doxycycline. For different porous chitosan/saccharomycetes microspheres, the adsorption for doxycycline increased with the increase in contact time, and the adsorption was quick in the first 90 min, and then the adsorption curves gradually became level. This is because with the increase in time, the adsorption sites on the adsorbent gradually decreased, and the adsorption rate and desorption rate were equal to reach the adsorption equilibrium, so the adsorption capacity would not increase [37].

The effect of the component proportion of the microspheres on the doxycycline adsorption behavior was significant. The adsorption capacity reached the maximum when the ratio of chitosan to saccharomycetes and silica gel was 1:2:3. It can be seen that the average pore volume and porosity of porous chitosan/saccharomycetes microspheres at the ratio of chitosan to saccharomycetes and silica gel of 1:2:3 was the largest, which produced a greater specific surface and porosity resulting in the increase in oxygen-containing functional groups, thus increasing the adsorption sites of doxycycline. In order to systematically analyze the adsorption and detection ability of porous chitosan/saccharomycetes microspheres for additives and volatiles in food and understand the adsorption mechanism of porous chitosan/saccharomycetes microspheres for different additives and volatiles, the adsorption and detection ability of porous chitosan/saccharomycetes microspheres for the additives and volatiles in food and the shelf life of foods treated by the porous chitosan/saccharomycetes microspheres will be described in the next manuscript.

## 4. Conclusions

Based on the characteristics of the silica gel dissolved in sodium hydroxide, porous chitosan/saccharomycetes adsorption microspheres were prepared by the titration and freeze-drying method, and the prepared porous chitosan/saccharomycetes microspheres were characterized. The method and the preparation process of the chitosan/saccharomycetes microspheres are green and pollution-free. Saccharomycetes can increase the roughness of the microsphere surface and improve the structure of the microsphere. Increasing the mass ratio of the silica gel can improve the compactness and porosity of the microspheres and form a porous spherical structure with uniform size and a good spherical shape. A good pore structure can improve the adsorption performance of microspheres, and saccharomycetes in the multi-aperture composite microsphere is an amorphous structure. Chitosan/saccharomycetes microspheres have good adsorption potential and the ability to adsorb and detect additives and volatiles in food or wastewater.

## Data Availability

The data presented in this study are available on request from the corresponding author.
